# Prognostic Performance of the Lactate-to-Ionized Calcium Ratio for In-Hospital Mortality in Adult Major Trauma: A Retrospective Emergency Department Cohort Study

**DOI:** 10.3390/diagnostics16142264

**Published:** 2026-07-20

**Authors:** Halil İbrahim Çimen, Mustafa Burak Sayhan, Eray Çeliktürk, Esin Seçgin Sayhan

**Affiliations:** 1 Department of Emergency Medicine, Trakya University Faculty of Medicine, 22030 Edirne, Turkey; 2 Department of Public Health, Trakya University Faculty of Medicine, 22030 Edirne, Turkey

**Keywords:** lactate-to-ionized calcium ratio, major trauma, in-hospital mortality, trauma severity indices, blood gas analysis, emergency department, prognosis

## Abstract

**Background/Objectives**: Early identification of adult major trauma patients at high risk of death remains challenging in the emergency department. Lactate reflects tissue hypoperfusion, whereas ionized calcium contributes to coagulation and cardiovascular function. This study evaluated the prognostic performance of the lactate-to-ionized calcium ratio (LiCa) for in-hospital mortality and compared it with established trauma scores. **Methods**: This single-center retrospective cohort study included adults with major trauma, defined as an Injury Severity Score (ISS) ≥ 16, who presented to a tertiary emergency department between May 2021 and April 2023. Demographic, clinical, laboratory, and outcome data were obtained from electronic records. LiCa was calculated as lactate divided by ionized calcium using the initial arterial blood gas sample obtained within 30 min of emergency department arrival. Factors associated with mortality were examined using multivariable logistic regression. Discrimination was assessed using receiver operating characteristic analysis and compared across LiCa, ISS, and Trauma and Injury Severity Score (TRISS) models. **Results**: A total of 236 patients were analyzed. In-hospital mortality occurred in 49 patients (20.8%). LiCa remained associated with in-hospital mortality after adjustment (adjusted odds ratio, 1.42; 95% confidence interval, 1.23–1.62; *p* < 0.001). LiCa showed good discrimination in this cohort, with an area under the curve of 0.93 (95% confidence interval, 0.88–0.97), compared with 0.94 for both TRISS 1995 and TRISS 2010 and 0.85 for ISS. At an exploratory threshold of ≥12.59, LiCa yielded 82% sensitivity, 94% specificity, a positive likelihood ratio of 12.72, and a negative likelihood ratio of 0.20. **Conclusions**: LiCa was independently associated with in-hospital mortality and showed good discrimination within this single-center retrospective cohort. As a rapidly available blood gas-derived measure, LiCa may provide exploratory adjunctive prognostic information at presentation, but it should not be interpreted as a replacement for validated trauma scoring systems. Prospective multicenter validation is needed before routine clinical use.

## 1. Introduction

Trauma remains a leading cause of mortality and long-term disability worldwide, particularly among individuals in the productive age group. Beyond its individual health consequences, trauma imposes a substantial burden on healthcare systems through increased resource utilization and organizational demands. The most recent Advanced Trauma Life Support (ATLS^®^) guidelines underscore the importance of early physiological assessment and a structured management approach in improving outcomes. Nevertheless, accurately identifying patients at high risk of mortality at the time of emergency department presentation continues to represent a critical clinical challenge [1].

The pathophysiological response to trauma is not solely determined by the extent of anatomical injury. Systemic inflammatory activation, microcirculatory impairment, cellular hypoxia, and metabolic dysregulation play central roles in determining prognosis. Elevated lactate levels, as markers of tissue hypoperfusion, have consistently been associated with increased mortality [2,3,4,5]. Similarly, reductions in ionized calcium have been associated with coagulopathy, increased transfusion requirements, and adverse outcomes in trauma patients [6]. However, these biomarkers are typically evaluated in isolation, potentially limiting their ability to reflect the integrated metabolic stress response following severe injury.

Early hemodynamic instability after trauma involves not only macrocirculatory compromise but also microcirculatory dysfunction, impaired cellular oxygen utilization, metabolic acidosis, and trauma-induced coagulation abnormalities. Lactate primarily reflects tissue hypoperfusion and anaerobic metabolism, whereas ionized calcium contributes to myocardial contractility, vascular tone, platelet function, coagulation cascade activation, and broader calcium-dependent cellular signaling processes [2,3,4,5,6,7]. Therefore, evaluating lactate and ionized calcium together may provide a more integrated representation of early metabolic and hemostatic stress than either parameter alone. The lactate-to-ionized calcium ratio may capture the combined effect of hypoperfusion-related lactate elevation and calcium-related physiological derangement during the early phase of major trauma [2,3,4,5,6,7].

Established trauma scoring systems, including ISS, NISS, RTS, and TRISS, are widely applied for mortality prediction [8,9,10,11,12,13,14,15]. Although these models integrate anatomical and physiological variables, they do not directly incorporate early biochemical manifestations of metabolic derangement. Recent studies suggest that biochemical markers may enhance early risk stratification; however, comparative data evaluating blood gas-derived ratios against conventional trauma scores remain limited [11,14,15,16,17]. This gap underscores the need to clarify the clinical utility of rapid, biochemically informed risk assessment strategies in trauma care.

Given its rapid availability and capacity to capture acute metabolic disturbances, blood gas analysis represents a practical and potentially valuable prognostic tool in the emergency setting. Nonetheless, the prognostic relevance of ratios derived from blood gas parameters and their comparative performance relative to established trauma scoring systems has not been fully elucidated.

In this study, we aimed to examine the association between blood gas-derived ratios obtained at emergency department presentation and in-hospital mortality among adult trauma patients, and to evaluate their predictive performance in comparison with commonly used and validated trauma scoring systems.

## 2. Materials and Methods

### 2.1. Study Design and Patient Selection

This was a single-center, retrospective observational cohort study conducted in the emergency department of a tertiary care hospital between 1 May 2021, and 30 April 2023. The study evaluated clinical and biochemical data of adult trauma patients. Ethical approval was obtained from the Institutional Clinical Research Ethics Committee of Trakya University Faculty of Medicine (Approval No: TÜTF-GOBAEK 2023/208; Date: 8 May 2023). The study was conducted in accordance with the Declaration of Helsinki. Due to the retrospective design, the requirement for informed consent was waived by the ethics committee. All data were anonymized prior to analysis.

**Figure 1:**
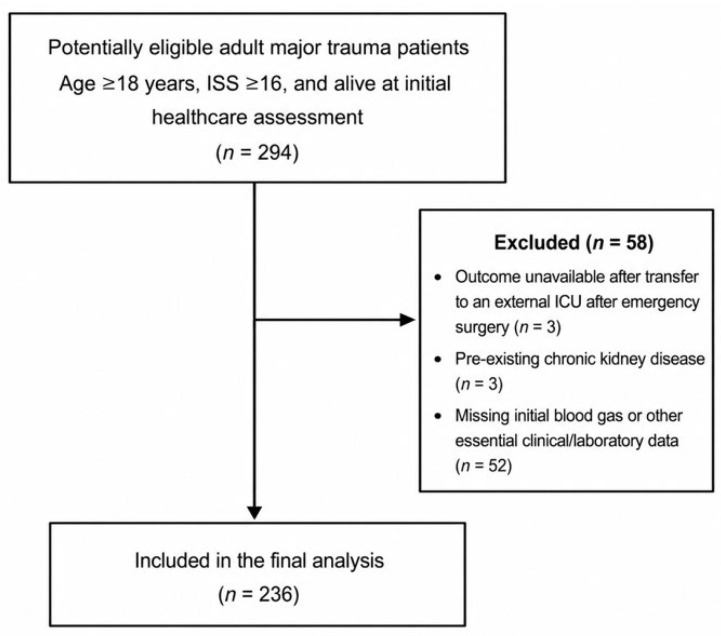
STROBE-style flow diagram of patient selection. ISS, Injury Severity Score; ICU, intensive care unit.

Adult patients (≥18 years) with major trauma, defined as an Injury Severity Score (ISS) ≥ 16, were eligible for inclusion. The ISS ≥ 16 threshold was selected because it corresponds to the conventional ISS > 15 definition commonly used to identify major or severely injured trauma populations in trauma research and registry-based studies (12,13). ISS values were calculated based on trauma registry records and the hospital’s electronic medical record system. Demographic characteristics, mechanism of injury, admission vital signs, and laboratory parameters were retrospectively extracted from the electronic hospital information system. Patients younger than 18 years, pregnant patients, individuals presenting with cardiac arrest on admission, and cases with incomplete initial blood gas or laboratory data were excluded from the study. During the study period, all trauma patients presenting to the emergency department were retrospectively screened. The patient-selection process is presented in a STROBE-style flow diagram in Figure 1.

A total of 294 potentially eligible adult major trauma patients were assessed, 58 were excluded, and 236 were included in the final analysis.

### 2.2. Data Collection

Demographic characteristics (age and sex), comorbidities, medication history, mechanism of injury, injury location and severity, admission vital signs, laboratory parameters, and clinical outcomes were retrospectively obtained from the hospital information management system and electronic medical records. Injury severity was assessed using the Abbreviated Injury Scale (AIS) and the Injury Severity Score (ISS). In addition, the New Injury Severity Score (NISS), Glasgow Coma Scale (GCS), Revised Trauma Score (RTS), and Trauma and Injury Severity Score (TRISS) models were calculated (7–10,14). The initial blood gas sample was defined as the arterial blood gas sample obtained within the first 30 min after emergency department arrival. Because all included blood gas samples were arterial, no adjustment or sensitivity analysis according to sample type was required. LiCa was calculated using the following formula: LiCa = lactate/ionized calcium. Lactate and ionized calcium were both recorded in mg/dL; therefore, LiCa was treated as a unitless ratio. Other blood gas-derived ratios were also calculated using this initial arterial blood gas measurement. Among these, the lactate-to-ionized calcium (LiCa) index was predefined as the primary variable of interest. Blood gas analyses were performed using a Radiometer ABL800 FLEX blood gas analyzer (Radiometer Medical ApS, Copenhagen, Denmark), according to the manufacturer’s instructions.

Information on prehospital interventions, exact pre-sampling fluid resuscitation volume, blood product administration before initial blood gas sampling, and calcium administration was not consistently available in the electronic records. Transfusion requirement during the early clinical course was recorded and summarized descriptively.

### 2.3. Outcomes

The primary outcome of the study was defined as in-hospital mortality. In-hospital mortality was defined as death occurring at any time during the index hospitalization related to the initial trauma admission. Patients were categorized into survivor and non-survivor groups according to mortality status. Baseline demographic, clinical, injury severity, transfusion, and laboratory characteristics were compared between survivors and non-survivors. The admitting department, emergency department length of stay, and total hospital length of stay were recorded as descriptive clinical variables only.

### 2.4. Statistical Analysis

Sample size calculation was based on a previously reported effect size evaluating the association between ISS and mortality in the literature [17]. All statistical analyses were performed using IBM SPSS Statistics for Windows, Version 26.0 (IBM Corp., Armonk, NY, USA). The distribution of continuous variables was assessed using the Kolmogorov–Smirnov test. Normally distributed variables were presented as mean ± standard deviation, whereas non-normally distributed variables were reported as median and interquartile range. Categorical variables were summarized as numbers and percentages. Baseline characteristics were additionally compared according to in-hospital mortality status. Between-group comparisons were performed using Student’s t-test or the Mann–Whitney U test for continuous variables and the Pearson chi-square test or Fisher’s exact test for categorical variables, as appropriate.

Univariable logistic regression analyses were performed to explore factors associated with in-hospital mortality. A multivariable logistic regression model was subsequently fitted using variables selected on the basis of their univariable associations and clinical relevance. The final model included age, hypertension, Injury Severity Score, and the lactate-to-ionized calcium ratio. Multicollinearity among variables included in the final multivariable model was assessed using variance inflation factor (VIF) and tolerance values. The multivariable model was restricted to variables available at emergency department presentation and selected according to clinical interpretability, event number, and potential overlap among trauma severity, physiological, and biochemical variables.

Transfusion requirement was not included in the main model because it represents a treatment- and resuscitation-related variable that may occur after initial assessment rather than an independent baseline predictor at presentation. Sample type was not included in the model because all blood gas samples were arterial. Lactate alone was not included in the same main model with LiCa because LiCa is mathematically derived from lactate and ionized calcium, which could introduce collinearity and reduce model interpretability. Given the retrospective design and the absence of a separate validation cohort, the multivariable model was considered exploratory. Associations were reported as odds ratios with 95% confidence intervals. Model fit was summarized using Cox and Snell R^2^ and Nagelkerke R^2^ statistics. The Hosmer–Lemeshow test was used as a supplementary goodness-of-fit assessment.

Discriminative performance was evaluated using receiver operating characteristic curve analysis, and areas under the curve were reported with 95% confidence intervals. Pairwise ROC comparisons between LiCa and other prognostic measures were performed using the DeLong test, and differences in AUC were reported as ΔAUC. The incremental value of LiCa was further assessed by comparing a base model including age, hypertension, and ISS with an extended model including age, hypertension, ISS, and LiCa. These nested models were compared using the likelihood ratio test, AUC, Nagelkerke R^2^, and Brier score. Calibration was assessed using the Hosmer–Lemeshow goodness-of-fit test and Brier score. Optimal cut-off values were identified using the Youden index. Because the LiCa cut-off was derived within the same cohort, the threshold was considered exploratory. Internal validation of LiCa discrimination and cut-off stability was performed using 1000 bootstrap samples. Sensitivity, specificity, positive predictive value, negative predictive value, and positive and negative likelihood ratios were calculated. All tests were two-sided, and *p* < 0.05 was considered statistically significant.

## 3. Results

### 3.1. Study Population

During the study period, the trauma registry was retrospectively reviewed to identify adult patients with major trauma who met the principal eligibility criteria. A total of 294 patients aged ≥ 18 years, with an ISS ≥ 16, and alive at the initial healthcare assessment were identified as potentially eligible. Of these, 3 patients were excluded because they were transferred to an external intensive care unit after emergency surgery and their in-hospital outcomes could not be ascertained, 3 because of pre-existing chronic kidney disease, and 52 because of missing initial blood gas or other essential clinical or laboratory data. The final analysis included 236 patients. In-hospital mortality occurred in 49 patients (20.8%), whereas 187 patients (79.2%) were classified as survivors. The median age was 41.5 (26–56) years, and the majority of patients were male (86.4%). At least one comorbidity was present in 25.4% of patients, and hypertension was present in 12.7%. Blunt trauma accounted for 78.0% of cases, while 22.0% sustained penetrating injuries. Baseline demographic, clinical, injury-severity, and laboratory characteristics stratified by in-hospital mortality status are presented in Table 1. Non-survivors were older and had greater anatomical injury severity, lower GCS and RTS values, higher lactate and LiCa values, and more frequent intubation and transfusion requirements than survivors.

### 3.2. Analysis of Factors Associated with Mortality

#### 3.2.1. Univariate Logistic Regression Analysis

Univariate logistic regression analysis was performed to identify variables associated with in-hospital mortality. Age, intubation status, ISS, NISS, GCS, RTS, sodium, chloride, lactate, ionized calcium, bicarbonate, base excess, albumin, and blood gas-derived ratios were significantly associated with in-hospital mortality in univariable analyses (all *p* < 0.05). Shock index was analyzed in patients with measurable admission vital parameters and was not significantly associated with in-hospital mortality.

The lactate-to-ionized calcium ratio (LiCa) demonstrated a strong positive association with mortality (OR = 1.40; 95% CI: 1.28–1.55; *p* < 0.001). Both TRISS 1995 and TRISS 2010 models showed significant inverse associations with mortality (*p* < 0.001 for both). Detailed results of the univariate regression analysis are presented in Table 2.

#### 3.2.2. Multivariate Logistic Regression Analysis

A multivariable logistic regression model including age, hypertension, ISS, and LiCa was fitted to evaluate their adjusted associations with in-hospital mortality. The model was based on 49 in-hospital mortality events and included four predictors, corresponding to approximately 12.3 events per variable. No relevant multicollinearity was detected among the final model variables; all VIF values were below 2.0, with the highest VIF observed for LiCa (VIF = 1.80). Age (adjusted OR = 1.07; 95% CI, 1.03–1.12; *p* < 0.001), hypertension (adjusted OR = 6.15; 95% CI, 1.34–28.19; *p* = 0.019), ISS (adjusted OR = 1.14; 95% CI, 1.04–1.24; *p* = 0.004), and LiCa (adjusted OR = 1.42; 95% CI, 1.23–1.62; *p* < 0.001) remained associated with in-hospital mortality after adjustment. The Cox and Snell R^2^ and Nagelkerke R^2^ values were 0.496 and 0.775, respectively. These pseudo-R^2^ measures provide supplementary information on model fit but should not be interpreted as measures of external predictive performance. The results of the multivariable analysis are presented in Table 3.

### 3.3. Model Performance

#### 3.3.1. Discrimination

**Figure 2:**
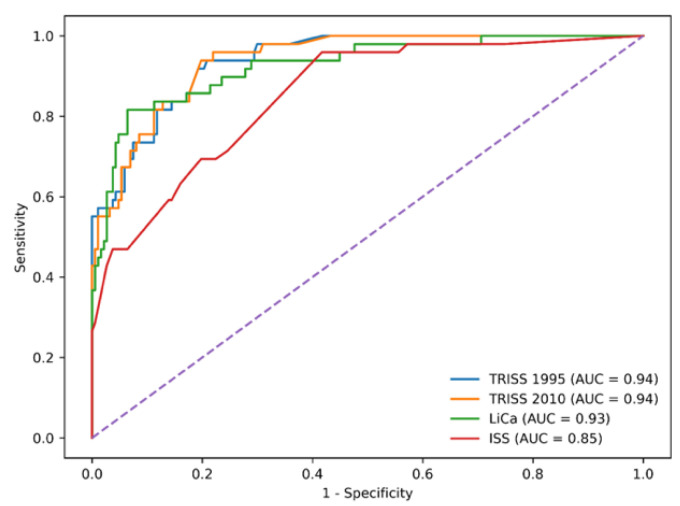
ROC curves demonstrating the discriminative performance of TRISS 1995, TRISS 2010, LiCa index, and ISS for in-hospital mortality.

The AUC was 0.94 (95% CI, 0.91–0.97) for both TRISS 1995 and TRISS 2010. LiCa yielded an AUC of 0.93 (95% CI, 0.88–0.97), whereas the AUC for ISS was 0.85 (95% CI, 0.79–0.91). The AUC for lactate alone was 0.92 (95% CI, 0.87–0.97). Pairwise ROC comparisons using the DeLong test showed that the AUC of LiCa was not significantly different from that of lactate alone (ΔAUC = 0.006; 95% CI, −0.006 to 0.018; *p* = 0.341), TRISS 1995 (ΔAUC = −0.013; 95% CI, −0.043 to 0.017; *p* = 0.401), or TRISS 2010 (ΔAUC = −0.014; 95% CI, −0.043 to 0.016; *p* = 0.362). However, LiCa demonstrated significantly higher AUC values than ISS, NISS, RTS, and GCS in pairwise comparisons. Detailed pairwise ROC comparisons are presented in Table 4. The corresponding ROC curves are presented in Figure 2.

TRISS models had the highest numerical AUC values, while LiCa showed a numerically similar AUC in this cohort. These findings should be interpreted together with pairwise ROC comparisons and should not be considered evidence that LiCa replaces established trauma scores.

#### 3.3.2. Cut-Off Values and Diagnostic Performance

The optimal cut-off value for TRISS 1995 was <0.90, with a sensitivity of 0.94 and specificity of 0.79. For TRISS 2010, the optimal threshold was <0.91, yielding a sensitivity of 0.94 and specificity of 0.80. For the LiCa index, the exploratory cut-off derived using the Youden index was ≥12.59, corresponding to a sensitivity of 0.82 and specificity of 0.94. The positive likelihood ratio was 12.72, and the negative likelihood ratio was 0.20. For ISS, the optimal cut-off was ≥24.50, with a sensitivity of 0.96 and specificity of 0.58. The cut-off values and corresponding sensitivity, specificity, predictive values, and likelihood ratios are summarized in Table 5.

#### 3.3.3. Calibration

The Hosmer–Lemeshow goodness-of-fit test was not statistically significant (*p* > 0.05), indicating that the test did not identify substantial lack of fit. The Brier score decreased from 0.092 in the base model to 0.053 in the extended model including LiCa, indicating lower overall prediction error after adding LiCa. However, these calibration findings should be interpreted cautiously because calibration plots, calibration slope, and external validation were not available.

#### 3.3.4. Incremental Model Performance and Internal Validation

Adding LiCa to the base model including age, hypertension, and ISS significantly improved model fit according to the likelihood ratio test (χ^2^ = 57.17; df = 1; *p* < 0.001). The AUC increased from 0.908 for the base model to 0.973 for the extended model including LiCa (ΔAUC = 0.065; 95% CI, 0.026 to 0.104; *p* = 0.001). Nagelkerke R^2^ increased from 0.554 to 0.771, and the Brier score decreased from 0.092 to 0.053. In 1000 bootstrap samples, the optimism-corrected AUC for LiCa was 0.926, which was similar to the apparent AUC. The bootstrap median LiCa cut-off was approximately 12.64, with a 95% interval of 8.21 to 13.23. Therefore, the LiCa threshold was interpreted as exploratory.

## 4. Discussion

The demographic and clinical characteristics of the study population were generally consistent with the existing trauma literature. The higher age observed among non-survivors and the persistence of an adjusted association between age and mortality support the well-established relationship between advanced age and adverse trauma outcomes. Reduced physiological reserve, increased comorbidity burden, and alterations in the systemic inflammatory response with aging may partly explain this relationship [1,3].

A higher rate of intubation was observed in the mortality group, likely reflecting the severity of physiological instability at presentation. However, intubation was not retained in the final multivariable model. This finding suggests that intubation may function more as a clinical marker of injury severity and acute physiological compromise rather than as a direct causal determinant of mortality [4].

The higher anatomical injury burden observed among non-survivors, particularly the greater frequency of head–neck injury, is consistent with the literature demonstrating the association between injury severity and clinical outcomes [18]. Injuries involving vital organ systems are known to play a decisive role in prognosis. Similar findings have been reported in studies evaluating the relationship between anatomical injury severity and mortality [11]. In addition, models integrating anatomical and physiological variables have been reported to demonstrate stronger performance in predicting mortality [10,15]. In this context, our findings support the prognostic value of multidimensional assessment approaches in trauma patients. In many prospective studies, anatomical and physiological scores such as ISS and RTS have been reported to demonstrate moderate to good discriminatory performance in predicting mortality; however, this performance has generally been lower than that of TRISS [3]. Similarly, in our study, the AUC values of ISS and RTS were found to be lower compared with TRISS. This difference may be related to the composite structure of TRISS, which incorporates anatomical injury severity, physiological parameters, and age [10,15]. While ISS primarily reflects anatomical injury burden and RTS represents the physiological status at presentation, neither score directly incorporates the biochemical components of early metabolic stress. This is consistent with previous evidence suggesting that ISS-based anatomical classification alone may be insufficient to fully characterize mortality risk in heterogeneous trauma populations [12,13]. In this context, the higher discriminatory performance of TRISS may be explained not by the inadequacy of individual scores, but rather by the more comprehensive risk assessment enabled by multidimensional data integration. Our findings are consistent with the literature suggesting that models combining anatomical and physiological variables may achieve stronger performance in mortality prediction.

Serum lactate is widely used as an early marker of tissue hypoperfusion in trauma patients. Ionized calcium, in contrast, plays a critical role in the coagulation cascade, myocardial contractility, and maintenance of vascular tone [14,17,19,20,21]. The combined evaluation of these two parameters may therefore reflect not only hypoperfusion but also the broader metabolic and electrolyte stress response that develops after trauma. Although the association between lactate and mortality has been well established in the literature, data on ratio-based approaches that simultaneously account for electrolyte balance remain limited [2].

In particular, the comparative performance of combined indices derived from blood gas analysis against established trauma scoring systems has not been sufficiently elucidated [14,22]. This gap highlights the need to clarify the clinical value of metabolically integrated biomarkers in trauma risk stratification.

In our study, LiCa remained independently associated with in-hospital mortality after adjustment. Its AUC of 0.93 was numerically close to the AUCs of 0.94 observed for the TRISS models. Pairwise ROC comparisons showed that the AUC of LiCa was not significantly different from that of lactate alone or the TRISS models, although LiCa demonstrated significantly higher AUC values than individual anatomical or physiological scores such as ISS, NISS, RTS, and GCS. In the incremental model analysis, adding LiCa to a base model including age, hypertension, and ISS improved model fit and discrimination. However, these findings should be interpreted as exploratory because the study was retrospective, single-center, and lacked external validation.

The Hosmer–Lemeshow test did not identify substantial lack of fit; however, comprehensive calibration assessment was limited. Pairwise ROC comparisons showed that the AUC of LiCa was not significantly different from that of lactate alone or the TRISS models. Therefore, LiCa should not be considered a replacement for existing trauma scores; rather, it may have potential adjunctive prognostic value during early trauma assessment. In emergency department settings, where rapid risk stratification at presentation is essential, its high specificity may provide additional support for clinical decision-making. Further prospective and multicenter validation studies are required before this index can be integrated into clinical algorithms and its generalizability across different patient populations can be established.

### 4.1. Clinical Implications

The LiCa ratio may have potential adjunctive prognostic value as a rapidly available blood gas-derived marker in adult patients with major trauma. Because both lactate and ionized calcium are commonly measured during initial trauma assessment, the LiCa ratio can be calculated without additional laboratory testing or cost. In the emergency department, a high LiCa ratio may help identify patients who warrant closer clinical assessment and monitoring. However, decisions regarding resuscitation intensity, trauma team activation, intensive care admission, or interhospital transfer should not be based on LiCa alone. The LiCa ratio should not be interpreted as a stand-alone decision-making tool or as a replacement for established trauma scoring systems; rather, it may serve as an adjunctive biochemical marker that complements anatomical and physiological assessment. Prospective multicenter validation, including assessment of clinical net benefit and risk reclassification, is required before this ratio can be incorporated into routine trauma algorithms.

### 4.2. Limitations

This study has several limitations. First, its single-center retrospective design may have introduced selection and information bias, and the findings may not be generalizable to trauma populations in other healthcare settings. Because detailed clinical and laboratory data for excluded patients were limited, a comprehensive comparison between included and excluded patients could not be performed; therefore, the possibility of selection bias cannot be fully excluded. Second, although a multivariable model was fitted, residual confounding cannot be excluded. Variable selection was informed by clinical relevance, event number, and potential overlap among trauma severity, physiological, and biochemical variables; however, model instability remains possible because the analysis was based on a single retrospective cohort. Although bootstrap internal validation was performed, no independent external validation cohort was available. Therefore, residual model optimism and overfitting cannot be fully excluded. Third, detailed prehospital management data, exact pre-sampling fluid resuscitation volume, blood product administration before initial blood gas sampling, and calcium administration were not consistently available in the electronic records. Because these interventions may influence lactate and ionized calcium levels, residual confounding related to early resuscitation cannot be excluded. Transfusion requirement was recorded and summarized descriptively, but it was not included in the main model because it represented a treatment- and resuscitation-related variable rather than a uniformly timed baseline predictor. Fourth, although pairwise ROC comparisons and bootstrap internal validation were performed, the LiCa cut-off was derived and evaluated within the same cohort. Therefore, threshold optimism cannot be fully excluded, and the reported cut-off should be considered exploratory until externally validated. In addition, although the Hosmer–Lemeshow test and Brier score were used to assess model performance, calibration plots and calibration slope were not available; therefore, calibration should be interpreted cautiously. Finally, decision curve analysis and net reclassification improvement analysis were not performed. Therefore, the clinical net benefit of LiCa and its potential impact on risk reclassification across different decision thresholds could not be assessed in the present study. Future prospective multicenter studies with external validation should evaluate whether LiCa improves clinical decision-making when added to established trauma scoring systems.

## 5. Conclusions

In this retrospective cohort of adult major trauma patients, LiCa remained associated with in-hospital mortality after adjustment for age, hypertension, and ISS. LiCa showed good discrimination, with an AUC numerically close to those of the TRISS models and lactate alone; however, it was not significantly different from these measures in pairwise ROC comparisons. These findings support LiCa as an exploratory, rapidly available adjunctive prognostic marker rather than a replacement for validated trauma scoring systems. Prospective multicenter studies with external validation are required before routine clinical implementation.

## Figures and Tables

**Table 1:** Baseline and clinical characteristics stratified by in-hospital mortality status.

Characteristic	Total Cohort (*n* = 236)	Survivors (*n* = 187)	Non-Survivors (*n* = 49)	*p*-Value
**Demographic characteristics and medical history**				
Age, years	41.5 (26–56)	39 (26–54)	50 (37–66)	0.004
Male sex	204 (86.4)	161 (86.1)	43 (87.8)	0.763
≥1 comorbidity	60 (25.4)	50 (26.7)	10 (20.4)	0.365
Hypertension	30 (12.7)	20 (10.7)	10 (20.4)	0.069
Anticoagulant use	5 (2.1)	3 (1.6)	2 (4.1)	0.278
Antiplatelet use	21 (8.9)	17 (9.1)	4 (8.2)	1.000
**Injury characteristics**				
**Injury type, ***n*** (%)**				
Blunt trauma	184 (78.0)	142 (75.9)	42 (85.7)	0.142
Penetrating trauma	52 (22.0)	45 (24.1)	7 (14.3)	
**Trauma mechanism, ***n*** (%)**				
In-vehicle traffic accident	58 (24.6)	51 (27.3)	7 (14.3)	0.025
Motorcycle accident	41 (17.4)	29 (15.5)	12 (24.5)	
Out-of-vehicle traffic accident	25 (10.6)	15 (8.0)	10 (20.4)	
Fall from height	45 (19.1)	35 (18.7)	10 (20.4)	
Assault/blunt violence	6 (2.5)	6 (3.2)	0 (0.0)	
Stab injury	29 (12.3)	27 (14.4)	2 (4.1)	
Firearm injury	23 (9.7)	18 (9.6)	5 (10.2)	
Other	9 (3.8)	6 (3.2)	3 (6.1)	
Head–neck injury	116 (49.2)	79 (42.2)	37 (75.5)	<0.001
Thoracic injury	175 (74.2)	142 (75.9)	33 (67.3)	0.222
Abdominal injury	83 (35.2)	71 (38.0)	12 (24.5)	0.079
Extremity/pelvic injury	79 (33.5)	62 (33.2)	17 (34.7)	0.839
**Trauma severity and admission physiology**				
ISS	25 (17–32)	20 (16–25)	34 (25–41)	<0.001
NISS	25 (18–34)	24 (17–29)	38 (34–43)	<0.001
GCS	15 (8–15)	15 (14–15)	3 (3–11)	<0.001
RTS	7.84 (5.97–7.84)	7.84 (7.11–7.84)	3.80 (0–6.90)	<0.001
* Shock index (available *n* = 221)	0.75 (0.62–0.92)	0.76 (0.63–0.88)	0.73 (0.57–1.18)	0.787
TRISS 1995 survival probability	0.94 (0.77–0.98)	0.96 (0.92–0.98)	0.17 (0.02–0.72)	<0.001
TRISS 2010 survival probability	0.95 (0.83–0.98)	0.96 (0.93–0.98)	0.42 (0.16–0.80)	<0.001
**Initial laboratory parameters**				
Lactate, mg/dL	27.0 (17.0–48.2)	24.0 (15.0–37.5)	73.0 (57.0–113.0)	<0.001
Ionized calcium, mg/dL	4.45 (4.19–4.62)	4.45 (4.21–4.60)	4.41 (3.93–4.71)	0.638
LiCa index	6.59 (3.96–10.99)	5.47 (3.46–8.40)	17.69 (13.08–27.03)	<0.001
Sodium, mmol/L	139 (137–141)	139 (137–140)	141 (138–143)	<0.001
Chloride, mmol/L	105 (103–107)	104 (102–107)	107 (104–110)	<0.001
Bicarbonate, mmol/L	20.8 (18.2–23.3)	21.7 (19.6–23.9)	14.8 (9.9–19.3)	<0.001
Base excess, mmol/L	−3.8 (−7.0 to −0.6)	−3.0 (−5.5 to 0.2)	−10.0 (−14.9 to −6.1)	<0.001
Albumin, g/dL	4.0 (3.7–4.4)	4.1 (3.7–4.4)	3.8 (3.2–4.2)	<0.001
Hemoglobin, g/dL	13.2 (11.8–14.3)	13.3 (11.9–14.6)	12.6 (10.0–13.9)	0.019
Platelet, 10^3^/µL	242 (192–291)	247 (198–294)	210 (137–281)	0.020
**Early clinical course**				
Intubation at admission	56 (23.7)	23 (12.3)	33 (67.3)	<0.001
Transfusion requirement	117 (49.6)	78 (41.7)	39 (79.6)	<0.001
ICU admission	103 (43.6)	82 (43.9)	21 (42.9)	0.901
Ward admission	55 (23.3)	53 (28.3)	2 (4.1)	<0.001
Emergency operation	61 (25.8)	52 (27.8)	9 (18.4)	0.179
Emergency department length of stay, hours	4.3 (2.3–7.3)	5.0 (2.8–7.8)	1.9 (1.1–4.0)	<0.001
Hospital length of stay, days	8.0 (4.0–17.2)	9.0 (5.0–18.5)	2.0 (1.0–16.0)	<0.001

**Footnote**: Data are presented as median (interquartile range) or number (%), as appropriate. Between-group comparisons were performed between survivors and non-survivors. Continuous variables were compared using the Mann–Whitney U test. Categorical variables were compared using Pearson chi-square or Fisher’s exact test, as appropriate. Blood gas variables were obtained from the initial arterial blood gas sample collected within the first 30 min after emergency department arrival. LiCa was calculated as lactate divided by ionized calcium. Because both lactate and ionized calcium were reported in mg/dL, LiCa was treated as a unitless ratio. Shock index was calculated as heart rate divided by systolic blood pressure and was summarized for patients with measurable systolic blood pressure. * Fifteen patients without measurable admission vital parameters were treated as missing for shock index analyses. ISS: Injury Severity Score; NISS: New Injury Severity Score; GCS: Glasgow Coma Scale; RTS: Revised Trauma Score; TRISS: Trauma and Injury Severity Score; LiCa: lactate-to-ionized calcium ratio; ICU: intensive care unit.

**Table 2:** Univariate logistic regression analysis for in-hospital mortality.

Variable	OR	95% CI	*p*-Value
**Age**	1.03	1.01–1.05	0.002
**Temperature**	0.08	0.03–0.21	<0.001
**Intubation (Yes)**	14.71	7.02–30.81	<0.001
**ISS**	1.17	1.11–1.22	<0.001
**NISS**	1.16	1.11–1.21	<0.001
**GCS**	0.77	0.72–0.83	<0.001
**RTS**	0.50	0.41–0.60	<0.001
**Shock index**	2.00	0.82–4.84	0.126
**Sodium**	1.29	1.16–1.43	<0.001
**Chloride**	1.15	1.07–1.23	<0.001
**Ionized calcium**	0.52	0.36–0.75	0.001
**Bicarbonate**	0.71	0.64–0.78	<0.001
**Lactate**	1.08	1.06–1.11	<0.001
**Base excess**	0.75	0.69–0.82	<0.001
**Albumin**	0.40	0.26–0.63	<0.001
**Lactate/iCa (LiCa)**	1.40	1.28–1.55	<0.001
**BE/iCa**	0.30	0.21–0.44	<0.001
**HCO_3_/iCa**	0.32	0.22–0.46	<0.001
**TRISS 1995**	0.002	0.0005–0.01	<0.001
**TRISS 2010**	0.0007	0.0001–0.01	<0.001

**Footnote**: OR: odds ratio; CI: confidence interval. Odds ratios were calculated for in-hospital mortality. Shock index was calculated as heart rate divided by systolic blood pressure and was analyzed only in patients with measurable admission vital parameters; patients without measurable admission vital parameters were treated as missing for shock index analyses. BE: base excess; iCa: ionized calcium; LiCa: lactate-to-ionized calcium ratio; HCO_3_: bicarbonate; TRISS: Trauma and Injury Severity Score.

**Table 3:** Multivariate logistic regression analysis for in-hospital mortality.

Variable	Adjusted OR	95% CI	*p*-Value
**Age**	1.07	1.03–1.12	<0.001
**Hypertension**	6.15	1.34–28.19	0.019
**ISS**	1.14	1.04–1.24	0.004
**Lactate/iCa (LiCa)**	1.42	1.23–1.62	<0.001

**Footnote**: Variables were selected according to clinical relevance, event number, and potential overlap among trauma severity, physiological, and biochemical variables. The model included four predictors for 49 in-hospital mortality events. Odds ratios (OR) were calculated for in-hospital mortality. Model fit was assessed using Cox and Snell R^2^ (0.496) and Nagelkerke R^2^ (0.775). Multicollinearity was assessed using VIF and tolerance values; all VIF values were below 2.0.

**Table 4:** Pairwise ROC comparisons between LiCa and other prognostic measures.

Comparison	LiCa AUC	Comparator AUC	ΔAUC	95% CI for ΔAUC	*p*-Value
**LiCa vs. lactate**	0.926	0.920	0.006	−0.006 to 0.018	0.341
**LiCa vs. ISS**	0.926	0.849	0.077	0.030 to 0.124	0.001
**LiCa vs. NISS**	0.926	0.846	0.080	0.031 to 0.129	0.001
**LiCa vs. RTS**	0.926	0.837	0.089	0.035 to 0.143	0.001
**LiCa vs. GCS**	0.926	0.838	0.087	0.027 to 0.148	0.005
**LiCa vs. TRISS 1995**	0.926	0.939	−0.013	−0.043 to 0.017	0.401
**LiCa vs. TRISS 2010**	0.926	0.939	−0.014	−0.043 to 0.016	0.362

**Footnotes**: AUC: area under the curve; CI: confidence interval; ΔAUC: difference in AUC. Pairwise comparisons were performed using the DeLong test. Positive ΔAUC values indicate higher AUC for LiCa.

**Table 5:** ROC analysis and prognostic performance for in-hospital mortality.

Variable	AUC (95% CI)	Cut-Off	Sensitivity	Specificity	PPV	NPV	LR (+)	LR (−)
**TRISS 1995**	0.94 (0.91–0.97)	<0.90	0.94	0.79	0.54	0.98	4.50	0.08
**TRISS 2010**	0.94 (0.91–0.97)	<0.91	0.94	0.80	0.55	0.98	4.74	0.08
**LiCa index**	0.93 (0.88–0.97)	≥12.59	0.82	0.94	0.77	0.95	12.72	0.20
**ISS**	0.85 (0.79–0.91)	≥24.50	0.96	0.58	0.38	0.98	2.30	0.07
**NISS**	0.85 (0.79–0.91)	≥30.50	0.78	0.77	0.47	0.93	3.37	0.29
**RTS**	0.84 (0.76–0.91)	<5.13	0.69	0.88	0.61	0.92	5.90	0.35
**GCS**	0.84 (0.77–0.91)	<11.50	0.78	0.82	0.54	0.93	4.39	0.27
**Shock index**	0.51 (0.39–0.64)	≥0.95	0.41	0.80	0.27	0.88	2.03	0.74
**Lactate**	0.92 (0.87–0.97)	≥49.50	0.84	0.91	0.71	0.96	9.20	0.18
**Bicarbonate**	0.83 (0.76–0.90)	<18.25	0.69	0.86	0.57	0.91	4.99	0.36
**Base excess**	0.84 (0.77–0.91)	<−5.80	0.80	0.78	0.49	0.94	3.63	0.26
**BE/iCa**	0.84 (0.77–0.91)	<−1.41	0.78	0.81	0.51	0.93	4.03	0.28
**HCO_3_/iCa**	0.78 (0.69–0.87)	<3.91	0.63	0.89	0.60	0.90	5.63	0.41

**Footnote**: Cut-off values were determined based on ROC curve analysis using the optimal threshold criterion. The direction of threshold values for continuous variables is indicated in the table (< or ≥). All analyses were performed for the in-hospital mortality outcome. AUC: Area under the curve; CI: Confidence interval; PPV: Positive predictive value; NPV: Negative predictive value; LR (+): Positive likelihood ratio; LR (−): Negative likelihood ratio. Shock index analyses were restricted to patients with measurable admission vital parameters; patients without measurable admission vital parameters were treated as missing. The LiCa cut-off was derived in the same cohort using the Youden index and should therefore be considered exploratory.

## Data Availability

The data supporting the findings of this study are available from the corresponding author upon reasonable request. The data are not publicly available due to privacy and ethical restrictions related to patient information.
